# Real-time geospatial analysis identifies gaps in COVID-19 vaccination in a minority population

**DOI:** 10.1038/s41598-021-97416-y

**Published:** 2021-09-13

**Authors:** Cici Bauer, Kehe Zhang, Miryoung Lee, Michelle Jones, Arturo Rodriguez, Isela de la Cerda, Belinda Reininger, Susan P. Fisher-Hoch, Joseph B. McCormick

**Affiliations:** 1 Department of Biostatistics and Data Science, School of Public Health, The University of Texas Health Science Center at Houston, 1200 Pressler St, Houston, TX USA; 2 Department of Epidemiology, Human Genetics and Environmental Science, School of Public Health, The University of Texas Health Science Center at Houston, Brownsville, TX USA; 3 City of Brownsville Public Health and Wellness, Brownsville, TX USA; 4 Department of Health Promotion and Behavior Sciences, School of Public Health, The University of Texas Health Science Center at Houston, Brownsville, TX USA

**Keywords:** Public health, Epidemiology, Population screening

## Abstract

COVID-19 vaccination is being rapidly rolled out in the US and many other countries, and it is crucial to provide fast and accurate assessment of vaccination coverage and vaccination gaps to make strategic adjustments promoting vaccine coverage. We reported the effective use of real-time geospatial analysis to identify barriers and gaps in COVID-19 vaccination in a minority population living in South Texas on the US-Mexico Border, to inform vaccination campaign strategies. We developed 4 rank-based approaches to evaluate the vaccination gap at the census tract level, which considered both population vulnerability and vaccination priority and eligibility. We identified areas with the highest vaccination gaps using different assessment approaches. Real-time geospatial analysis to identify vaccination gaps is critical to rapidly increase vaccination uptake, and to reach herd immunity in the vulnerable and the vaccine hesitant groups. Our results assisted the City of Brownsville Public Health Department in adjusting real-time targeting of vaccination, gathering coverage assessment, and deploying services to areas identified as high vaccination gap. The analyses and responses can be adopted in other locations.

## Introduction

COVID-19 vaccination is being rapidly rolled out in the US and many other countries, but barriers in disadvantaged and highly vulnerable communities are significant. In this analysis, we reported the effective use of real-time geospatial analysis to identify barriers and gaps in COVID-19 vaccination in a minority population to inform rollout strategies and vaccination campaigns. The study region was the City of Brownsville (COB, population 182,781), the largest city within Cameron County (population 421,750) in South Texas on the US-Mexico Border^1^. Over 90% of the population is Mexican–American, with high prevalence of Type 2 diabetes and obesity (27% and 50%), respectively leading risk factors to nearly twice the national mortality from COVID-19^2–4^. The population is historically underserved and socioeconomically disadvantaged with limited healthcare access ^2^.

Despite the disproportionally high impact of COVID-19 on minorities and underserved populations, a recent study reported lower COVID-19 vaccination coverage in socially vulnerable populations ^5^. It is therefore crucial to provide fast and accurate assessment of vaccination coverage to identify and remedy local gaps. Since the COVID-19 vaccine first became available in COB in early February 2021, researchers from the University of Texas School of Public Health at Brownsville have been working closely with the COB Public Health Department, to provide real-time geospatial analysis to assess vaccination gaps. We developed a real-time evaluation to quantify vaccination needs, as well as identify and locate gaps at census tract level. Our assessment has provided the COB Public Health Department with the needed information to reach vulnerable groups, improve overall vaccination uptake, and decide where to deploy their ‘boots-on-the-ground’ vaccination campaigns.

## Data and materials

### Study region and population

The minority population in the COB has been disproportionally affected by COVID-19, with cumulative COVID-19 case rate of 9.13%, case fatality rate (COVID-19 death among COVID-19 cases) of 3.42% and mortality rate (COVID-19 death among total population) of 0.31%, between March 19th, 2020 (first case reported from COB) and March 13th, 2021, reported from Cameron County at the time of this analysis ^6^. All three rates were much higher than the national cumulative case rate of 8.9%, case fatality rate of 1.82% and overall mortality rate of 0.16% ^7^. This population is also among the most vulnerable populations, with the social vulnerability index (SVI) for Cameron County ranked among the lowest 5% in the US (i.e., SVI rank 96.7%) ^8^. Our analysis included all 48 census tracts that COB encompasses.

### COVID-19 vaccination rollouts

COB Public Health Department functioned as a local COVID-19 vaccination hub provider held four mass vaccinations clinics, with first dose clinics conducted on February 5th, February 13th, February 26th, 2021, and a second dose clinic on March 5th, 2021. The Moderna mRNA-1273 COVID-19 vaccine (Moderna TX, Inc, Cambridge, MA) was administered during these mass vaccination clinics. COB also held a small clinic the week of March 9th, 2021 with the Janssen (Ad.26.COV2.S) vaccine (Janssen Biotech, Inc, a Janssen Pharmaceutical company, Johnson & Johnson; New Brunswick, NJ), at the Fire Station for home-bound individuals and transportation-restricted individuals. The initial rollout of the COVID-19 vaccination eligibility was based on Texas Department of State Health Services vaccine rollout plan for front-line health workers (Phase 1A), those aged 65 years and older, and/or those aged 16 years and older with at least one medical condition with increased COVID-19 risk (Phase 1B). COVID-19 vaccination data in this report were obtained from the Texas Immunization Registry database (ImmTrac2) and encompass all those vaccinated in the city clinics since February 5th through March 13th, 2021. These data were used to assess vaccination coverage up to March 13th during the initial phase. We calculated the COVID-19 vaccination coverage as the population that has received at least one dose of vaccination per 10,000 population, for each census tract within the study region and during this initial rollout phase.

### COVID-19 vaccination gap

We followed the COVID-19 Vaccination Planning to assess local COVID-19 vaccination gaps ^9^. We first ranked the census tracts by their vaccination priority from the lowest to the highest, and then ranked the census tracts by their actual vaccinations coverage rates also from the lowest to the highest. The difference between the two ranks was then used to quantify the vaccination gap.

To assess the COVID-19 vaccination priority, different metrics can be used, and each may focus on a different aspect of COVID-19, such as the impact, social vulnerability, or priority population eligible for COVID-19 vaccination. Here we considered and compared the following four priority assessments. In Approach 1, we used census tract cumulative COVID-19 case rate reported above, and areas with high rate were considered priority. Approach 2 used SVI, and so areas with higher SVI were ranked as higher priority. Approach 3 used the census tract percentage of population aged 65 and older, so that areas with higher percentage of elderly were designated as higher priority. This approach accounted for the vaccination eligibility in the initial rollout phase, as the higher priority was given to the elderly. Finally in Approach 4, we used the COVID-19 community vulnerability index (CCVI) created by Surgo Ventures ^10^. CCVI covered seven themes of COVID-19 risk factors including socioeconomic status, work environment, ethnicity and healthcare system. We averaged the theme-specific scores to obtain an overall CCVI composite score, which ranged from 0 to 1 with 0 the lowest vulnerability and 1 the highest. The overall composite score was used to rank the census tracts for vaccine priority.

The COVID-19 vaccination gap was assessed by each of the four approaches. Census tracts were identified as having vaccination gap if the rank of vaccination coverage rates was lower than the rank of priory; otherwise, they were identified as no gap. Among the census tracts with vaccination gaps, we further classified them into high, medium and low gap group by dividing the rank difference to 3 equal size groups. All analyses were performed in R Studio ^11^. To create the maps presented in this analysis, we first obtained the GIS shapefiles from 2019 TIGER/Line shapefiles from the U.S. Census Bureau^12^, and used R package “tmap” to create the choropleth maps^13^.

## Results

Table 1 presents the summary statistics for all COVID-19 vaccines administrated in COB, stratified by age, gender, ethnicity and dosage. From February 5th to March 13th, 2021, a total of 3743 (1.6% of the total population; 2.2% of the population aged 16 years and older) people have been vaccinated (i.e., received at least one dosage), with 2176 fully vaccinated. Of those vaccinated, 1,711 (45.7%) were aged 65 and older. We summarized the vaccinated individuals by their age group (under 20, 20–39, 40–64 and 65 and greater), sex and ethnicity.

**Table 1:** Summary of COVID-19 vaccination in City of Brownsville between February 5th, 2021 (COVID-19 vaccination first became available to the region) and March 13th, 2021. At least one dose refers to either completing one or two doses of Moderna mRNA-1273 COVID-19 vaccine, or one dose of Janssen (Ad.26.COV2.S) vaccine. Complete dose refers to either completing two doses of Moderna mRNA-1273 COVID-19 vaccine, or one dose of Janssen (Ad.26.COV2.S) vaccine.

Variable	At least one dose (n = 3743)	Complete dose (n = 2176)
**Age Group**		
Under 20	59 (1.6%)	11 (0.5%)
Between 20 to 39	681 (18.2%)	218 (10.0%)
Between 40 to 64	1292 (34.5%)	537 (24.7%)
65 and greater	1711 (45.7%)	1410 (64.8%)
**Gender**		
Female	2240 (59.8%)	1289 (59.2%)
Male	1495 (39.9%)	869 (39.9%)
Missing	8 (0.3%)	18 (0.9%)
**Ethnicity**		
Hispanic or Latino	2722 (72.7%)	1790 (82.3%)
Not Hispanic or Latino	169 (4.5%)	154 (7.1%)
Missing	852 (22.8%)	232 (10.7%)

**Figure 1:**
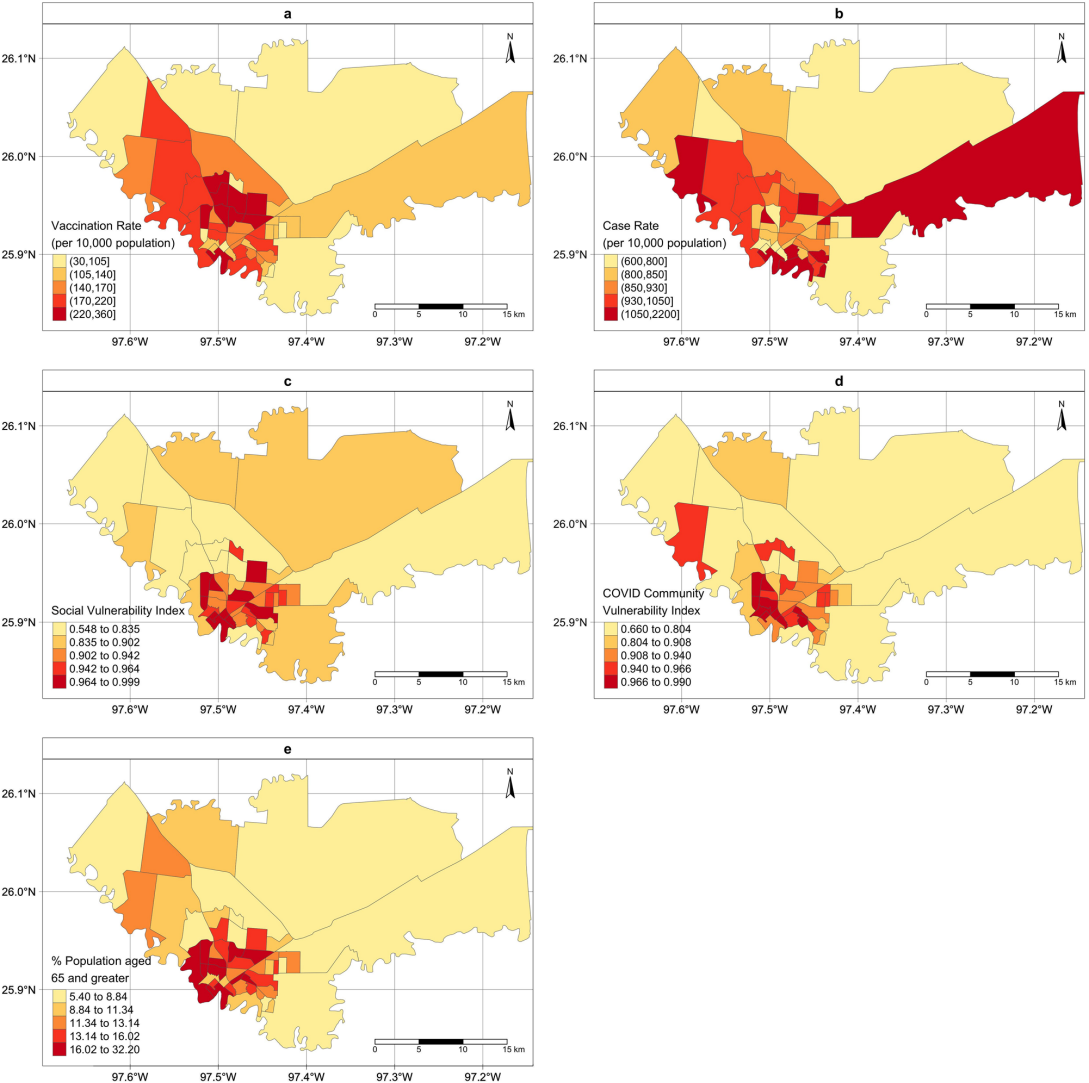
Maps of the census-tract level vaccination rate and vaccination priority in City of Brownsville, TX. (**a**): Vaccination rate per 10,000 population (February 5th–March 13th, 2021); (**b**): Case rate per 10,000 population (March 18th, 2020–March 13th, 2021). (**c**): social vulnerability index (SVI) ranked from 0 to 1; (**d**): COVID community vulnerability index (CCVI) ranked from 0 to 1; (**e**): percentage of population with 65 years old and over. The choropleth maps were created by the authors using R Studio (version 1.2.1335). URL http://www.rstudio.com/, and R package *tmap.*

Figure 1 presents the census tract level of vaccination coverage rate between February 5th and March 13th, 2021 (**a**), cumulative COVID-19 case rate between March 18th, 2020 and March 13th, 2021 (**b**), CDC SVI for year 2018 (**c**), percentage of population 65 years old and above (**d**) and CCVI composite score (**e**). These maps present somewhat different spatial patterns and identify different sets of census tracts as the priority areas.

**Figure 2:**
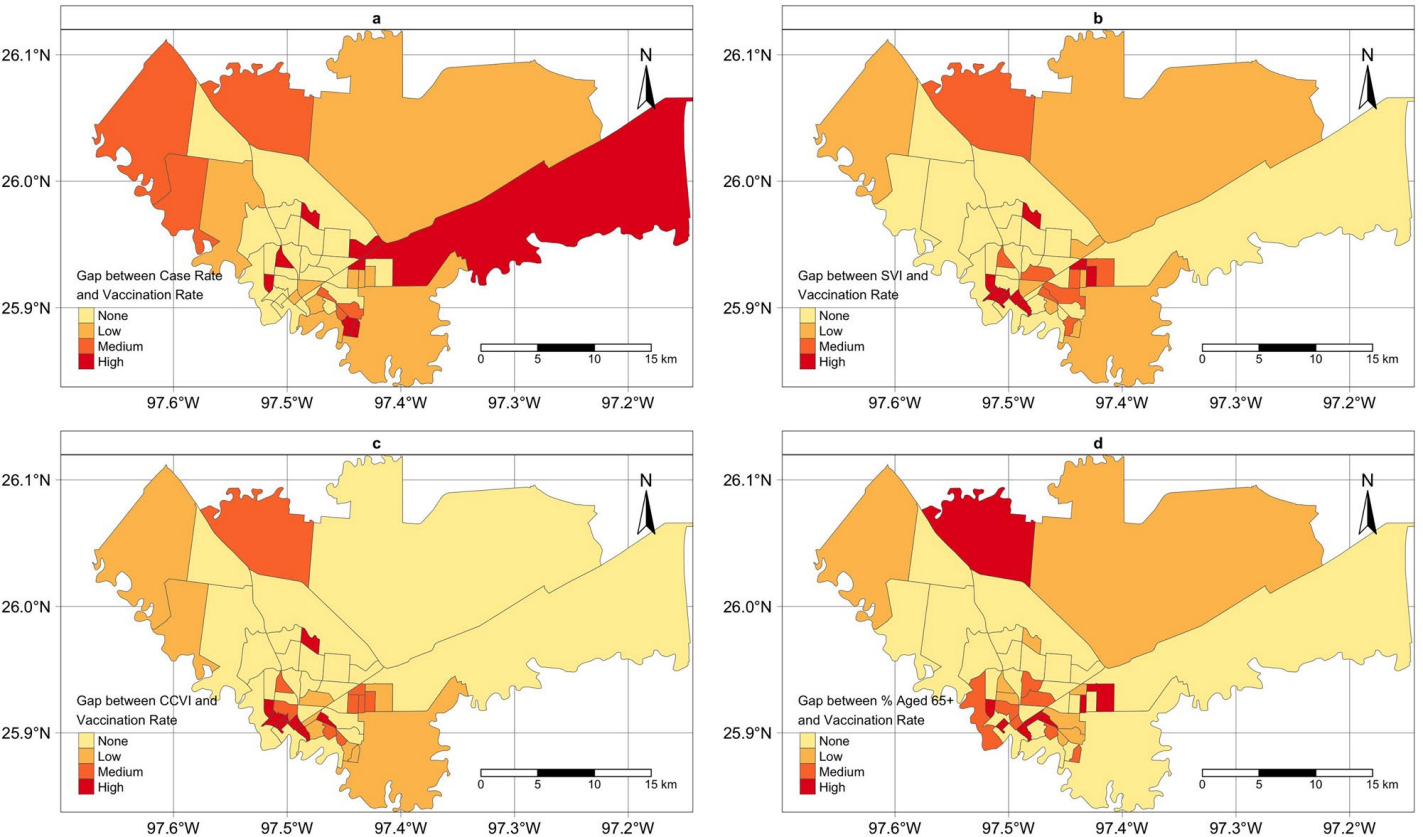
Maps of census tracts identified with no-gap/low/median/high vaccination gap in City of Brownsville, TX. Each panel corresponds to a different approach when assessing the gap proposed in this analysis. The choropleth maps were created by the authors using R studio (version 1.2.1335). URL http://www.rstudio.com/*,* and R package *tmap.*

Figure 2 presents the maps of census tracts identified as no-gap/low/median/high gap. Each panel corresponds to one of the four approaches described above for assessing the gap. Some areas were identified as high gap areas by all four approaches, while some difference was also noticed. We noted some rural areas on the outskirts of the city were identified as high gap area only when using the cumulative case rate to assess the gap. Areas in the city downtown were consistently identified as high gap areas.

The analysis has allowed COB Public Health Department to strategically target areas with high vaccination gaps through their “boots on the ground” campaign, while considering the population characteristics in these areas (e.g., age or primary language spoken at home). For example, COB Public Health Department has developed and implemented a vaccination messaging approach utilizing door-to-door outreach in both English and Spanish languages in identified high gap areas with older populations, with additional messaging via the city’s social media platforms and the weekly COVID update targeting high gap areas with higher young populations^14^.

## Discussion

In this analysis, we reported the first phase of COVID-19 vaccination rollout in the COB between February 5th, 2021 and March 13th, 2021. We developed four rank-based approaches to evaluate the vaccination gap, which considered COVID-19 impact, population vulnerability and vaccination eligibility. The rank-based approach, despite being simple, can accommodate the fast-changing dynamics of COVID-19 vaccination. For example, starting March 29th, 2021, vaccination eligibility for COVID-19 vaccination in Texas will include all population aged 16 years and older. Eligibility criteria should be accounted for when assessing vaccination gaps. As COVID-19 vaccine distribution plans evolve, together with COVID-19 herd immunity accumulating, we need a flexible approach such as the ones developed in this analysis to enable rapid vaccine strategy adaptations.

Real-time geospatial analysis to identify vaccination gaps is critical to increase vaccination uptake in the vulnerable and vaccine hesitant groups^15^. Our analyses have assisted the COB Public Health Department in adjusting real-time targeting of vaccination, gathering coverage assessment, and the deployment of services to areas with the largest gaps. Along with low education and low income levels, Hispanics showed higher COVID-19 vaccine hesitancy compared with their counterparts^15^. Our real-time geospatial analysis incorporating SVI, for example, would provide the information on vaccine hesitant groups overlapping with high SVI. The effective use of real-time geospatial analysis can assist local public health departments, particularly those with limited resources, to develop effective, as well as cultural and language appropriate strategies to reach communities and increase vaccination uptake.
